# The innate immune repertoire in Cnidaria - ancestral complexity and stochastic gene loss

**DOI:** 10.1186/gb-2007-8-4-r59

**Published:** 2007-04-16

**Authors:** David J Miller, Georg Hemmrich, Eldon E Ball, David C Hayward, Konstantin Khalturin, Noriko Funayama, Kiyokazu Agata, Thomas CG Bosch

**Affiliations:** 1 ARC Centre of Excellence in Coral Reef Studies and Comparative Genomics Centre, James Cook University, Townsville, Queensland 4811, Australia; 2 Zoological Institute, Christian-Albrechts-University Kiel, Olshausenstrasse, 24098 Kiel, Germany; 3 ARC Centre for the Molecular Genetics of Development, Research School of Biological Sciences, Australian National University, Canberra ACT 2601, Australia; 4 Department of Biophysics, Kyoto University, Kitashirakawa-Oiwake, Sakyo-ku, Kyoto 606-8502, Japan

## Abstract

**Background:**

Characterization of the innate immune repertoire of extant cnidarians is of both fundamental and applied interest - it not only provides insights into the basic immunological 'tool kit' of the common ancestor of all animals, but is also likely to be important in understanding the global decline of coral reefs that is presently occurring. Recently, whole genome sequences became available for two cnidarians, *Hydra magnipapillata *and *Nematostella vectensis*, and large expressed sequence tag (EST) datasets are available for these and for the coral *Acropora millepora*.

**Results:**

To better understand the basis of innate immunity in cnidarians, we scanned the available EST and genomic resources for some of the key components of the vertebrate innate immune repertoire, focusing on the Toll/Toll-like receptor (TLR) and complement pathways. A canonical Toll/TLR pathway is present in representatives of the basal cnidarian class Anthozoa, but neither a classic Toll/TLR receptor nor a conventional nuclear factor (NF)-κB could be identified in the anthozoan *Hydra*. Moreover, the detection of complement C3 and several membrane attack complex/perforin domain (MAC/PF) proteins suggests that a prototypic complement effector pathway may exist in anthozoans, but not in hydrozoans. Together with data for several other gene families, this implies that *Hydra *may have undergone substantial secondary gene loss during evolution. Such losses are not confined to *Hydra*, however, and at least one MAC/PF gene appears to have been lost from *Nematostella*.

**Conclusion:**

Consideration of these patterns of gene distribution underscores the likely significance of gene loss during animal evolution whilst indicating ancient origins for many components of the vertebrate innate immune system.

## Abstract 2

Analysis of genomic resources available for cnidarians revealed that several key components of the vertebrate innate immune repertoire are present in representatives of the basal cnidarian class Anthozoa, but are missing in Hydra, a member of the class Hydrozoa, indicating ancient origins for many components of the innate immune system.

## Background

The innate immune system is the first line of defense against pathogens, and in non-chordates is assumed to be the sole means by which any non-self cells are detected and either killed or contained [1]. Innate immunity in vertebrates is essentially a two-tier system consisting on one hand of phagocyte activation by the interaction of specialized surface receptors with pathogens or pathogen-derived components, and on the other of the direct opsonization and lysis of pathogens via the complement cascade. Whilst the vertebrate innate immune system has been the subject of intense investigation and is relatively well understood, studies of invertebrate immunity, which have focused primarily on the arthropods *Drosophila *and various horseshoe crab species [2-4], have revealed some striking similarities. For example, in both *Drosophila *and vertebrates, the Toll/Toll-like receptor (TLR) mediates the activation of appropriate response genes to microbial challenge [5,6].

Toll and the TLRs are transmembrane proteins with a characteristic domain structure consisting of an extracellular amino-terminal domain containing leucine-rich repeats (LRRs) responsible for pattern recognition and an intracellular Toll interleukin receptor (TIR) domain that mediates signal transmission. Although the Toll and TLR families of arthropods and mammals are thought to have independently diversified [7,8], all Tolls and TLRs signal via a common pathway that is conserved between *Drosophila *and mammals. The ultimate step in this pathway is translocation of nuclear factor (NF)-κB or its fly counterpart (the Dif/Rel heterodimer) into the nucleus, where it stimulates transcription of appropriate response genes. The immune repertoire of the horseshoe crab *Carcinoscorpius *includes a complex complement pathway that has both opsonic and lytic effector functions [9]. Horseshoe crab complement C3 is functionally homologous with mammalian C3, mediating phagocytosis of bacteria (by hemocytes) in a strikingly similar manner.

Whilst these specific studies imply that at least some innate immune mechanisms have been conserved, broader comparative studies highlight the extent of gene loss and divergence in various metazoan lineages. For example, although *Carcinoscorpius *clearly uses a vertebrate-like complement system, none of the central components of the cascade (C2, C3, C4, C5) are encoded by the genomes of the ecdysozoans *Drosophila*, *Caenorhabditis *or *Anopheles*. Moreover, the sole Toll/TLR in *Caenorhabditis elegans *and *C. brigssae *is not known to function in the context of immunity, nor does that reported in the horseshoe crab *Tachypleus tridentatus *[10]. There are also important differences between the Toll/TLR systems of *Drosophila *and mammals. For example, some mammalian TLRs themselves act as pattern recognition receptors (PRRs) upon microbial challenge, whereas in fly this is not the case [11]. Moreover, whereas most of the ten or so vertebrate TLRs function primarily in immunity, only one of the nine fly (and ten mosquito) Tolls functions in this context. The others play a role in development [10], most famously in controlling differentiation in the dorsal/ventral axis.

**Figure 1:**
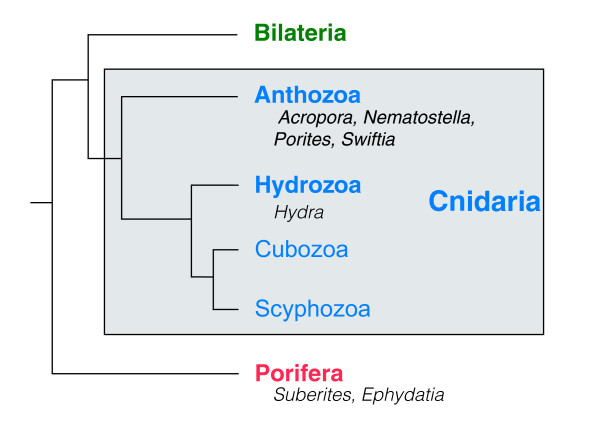
Relationships at the base of the Metazoa. Cnidarians are amongst the simplest animals at the tissue grade of organization, and are often regarded as the closest outgroup to the Bilateria. Within the Cnidaria, the class Anthozoa is basal, whereas the Hydrozoa is derived. The sponges (Porifera) are unquestionably animals, but represent a lower level of organization. The affinities and relationships of genera mentioned in the text are indicated.

The significance of gene loss in animal evolution has recently been brought into focus by preliminary expressed sequence tag (EST) and genomic analyses of some 'basal' animals (Figure 1), particularly the anthozoan cnidarians *Acropora millepora *and *Nematostella vectensis *[12,13] and the planarian *Dugesia japonica *[14]. Paradoxically, the genomes of these morphologically simple animals contain many genes previously thought to have evolved much later in the context of vertebrate complexity, and most of the complexity of signaling pathways and transcription factors associated with higher animals is represented in the anthozoan datasets [13,15-17]. In contrast to *Drosophila *and *Caenorhabditis*, which have undergone substantial gene loss, for at least some groups of genes *Acropora *and *Nematostella *appear to have preserved much of the genetic complexity of the common metazoan ancestor. For example, whereas fly and worm have each lost approximately half of the ancestral Wnt complement, all but one of the 12 known Wnt subfamilies is represented in *Nematostella *[15]. The emerging cnidarian EST and genomic datasets are, therefore, potentially highly informative with respect to the ancestral immunological repertoire. In addition to this basic evolutionary significance, understanding the bases of cnidarian immunity is of major applied significance in light of the dramatic decline in coral health that has occurred on a global scale over the past 20 years. Increasing human activity in coastal zones throughout the world has led to declines in water quality with increased sediment, nutrient and heavy metal concentrations. These have all had detrimental effects on corals with an associated increase in the prevalence of disease, and perhaps led to some new diseases, although this is less certain, as coral diseases are very poorly understood. Most are named for their symptoms, for example, 'white band disease', 'black band disease', and 'rapid wasting syndrome' and causative agents are frequently unknown. In the face of this uncertainty it is important that coral defense mechanisms should be better understood.

Although cnidarians have no specialized immune cells, at least some display highly specific allorecognition characteristics. Allorecognition, xenorecognition, and killing mechanisms have been demonstrated in several hydrozoans [18-20] and anthozoans [21-23]. Allorecognition is thought to protect colonial cnidarians from fusion with genetically different individuals and to prevent germ line parasitism. The effector mechanisms range from contact avoidance involving chemical sensing, to barrier formation, or usage of nematocysts. For example, the sea anemone *Anthopleura xanthogrammmica *will 'tolerate' adjacent clonal individuals, but will attempt to 'reject' heterogenic clones with which it comes into contact [24,25]. In the anthozoans, *Stylophora pistillata *and *Montipora verrucosa *branches within one colony will readily fuse while branches of genetically different individuals never undergo fusion [22,26,27]. Fusion of two conspecific individuals is occasionally referred to as 'natural transplantation'. Observations in sea anemones (*Anthopeura elegantissima*, *Phymactis clematis*) and gorgonians (*Eunicella stricta*) indicate that individual colonies possess unique sets of histocompatibility elements, which are recognized as nonself by all other conspecific colonies [23,28]. In Hydrozoa, the same phenomenon was reported for *Millepora dichotoma *[29] and studied in great detail in the colonial marine hydroid *Hydractinia echinata *[30]. *Hydractinia *in fact was among the first invertebrates shown to display a genetically based system of intolerance against allogenic tissue: for more than 50 years it has been known that allorecognition and the inability to fuse stolons of different colonies is under the control of one polymorphic gene [31,32]. Recent efforts using defined genetic lines of the hydroid *Hydractinia symbiolongicarpus *have confirmed this and shown that the single chromosomal region contains at least two loci [33].

The availability of whole genome sequences of two basal cnidarians at respectably high levels of redundancy - the hydrozoan *Hydra magnipapillata *(>6-fold coverage) and the anthozoan *Nematostella vectensis *(>7.6-fold coverage [17]) - together with large-scale EST datasets for these and for the coral *Acropora millepora *potentially offer new perspectives on the origins of mammalian immune functions. Here we report the results of a screen of the available genomic and EST resources for the cnidarian counterparts of key components of the vertebrate innate immune repertoire.

## Results

### The toll receptor and other proteins containing the TIR domain

**Figure 2:**
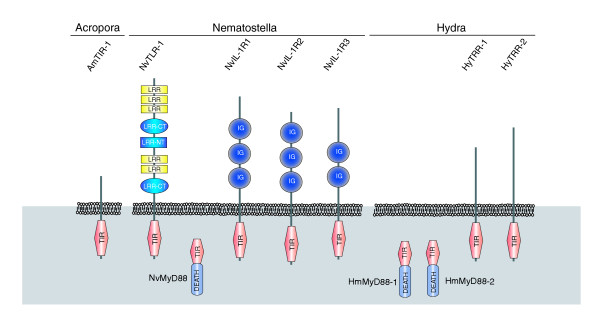
Summary of domain structures of TIR domain-containing proteins identified in selected Cnidaria.

Searching the *Hydra *predicted protein collection using the available hidden Markov models (HMMs) identified only four TIR domain-containing proteins, two of which are clearly related to MyD88, which functions downstream of TLRs (Table 1). Consistent with their assignment as MyD88 family members, both of these *Hydra *proteins also contain the characteristic DEATH domain. The two other *Hydra *TIR proteins are atypical transmembrane proteins in having relatively short extracellular domains that are devoid of the LRR domains that characterize Toll and the TLRs (Figure 2). cDNAs encoding these proteins have previously been isolated by the Bosch laboratory (unpublished data) and their functions are presently under investigation; these proteins are known as HyTRR-1 and HyTRR-2. Surprisingly, extensive searching of the *Hydra *genome and all available EST/cDNA resources failed to identify any proteins having the canonical Toll/TLR structure, characterized by possession of both LRR and TIR domains.

**Figure 3:**
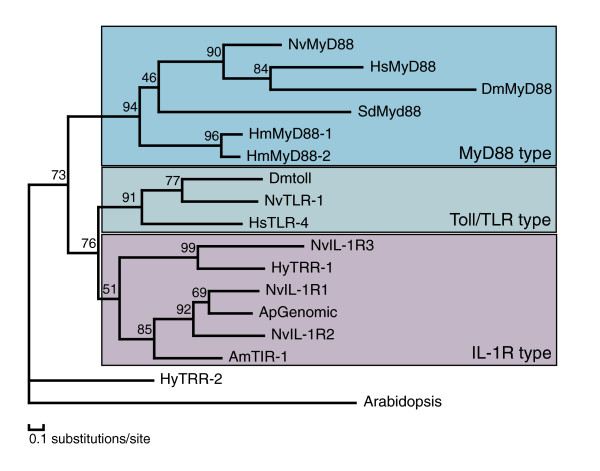
Phylogenetic analysis of cnidarian TIR sequences in comparison to a selection of TIR domains from other species. The maximum likelihood (ML) tree shown is the result of analysis of an HMM-based alignment of TIR domains. A number of the TIR sequences identified and discussed in the text are incomplete due to the presence of introns of unknown size, and hence were not included in the phylogenetic analyses. Three clades are resolved by these analyses, corresponding to the TIR domains characteristic of the 'MyD88-type', 'Toll/TLR-type' and 'IL-1R-type'. In addition to the TIR domain, the first of these types contains a death domain and the second contains multiple LRRs. Like the mammalian receptors for interleukin 1, the three *Nematostella *proteins falling into the third clade each also contain multiple immunoglobulin domains. Note that HyTRR1 does not contain such domains and that it is not yet clear whether either of the *Acropora *proteins does. The *Acropora *sequences included in the analysis were predicted from *A. palmata *genomic clones (ApGenomic) and from an *A. millepora *cDNA clone (AmTIR-1). *Hydra *lacks a canonical Toll/TLR, having only two MyD88 genes and the two sequences known as TRR-1 and TRR-2; *H. magnipapillata *and *N. vectensis *sequences are indicated by the prefixes Hy and Nv, respectively. Reference sequences: HsMyD88, human MyD88 (SwissProt:Q99836); DmMyD88, fly MyD88 (GenBank:AAL56570); SdMyD88, *Suberites *MyD88 (EMBL:CAI68016); Dmtoll, fly Toll (SwissProt:P08953); HsTLR4, human TLR4 (EMBL:CAD99157); *Arabidopsis *(GenBank:AAN28912).

Whereas only four TIR proteins are present in *Hydra*, substantially more could be identified amongst the predicted proteins from *Nematostella *using HMM-based search methods. Five of them were sufficiently complete to be included in the analyses presented here. These include a single MyD88 homolog (NvMyD88) and a protein (NvTLR-1) clearly related to members of the Toll/TLR family (Figure 2). Whereas the mammalian TLRs, and some members of the fly Toll/TLR family, have only a carboxy-terminal cysteine-rich motif flanking the LRRs proximal to the membrane, *Nematostella *NvTLR-1 is predicted to contain both carboxy- and amino-terminal-flanking cysteine-rich motifs in the extracellular part of the protein (Figure 2). This suggests that fly and anemone Toll more closely reflect the ancestral domain structure than do the mammalian TLRs. Moreover, a phylogenetic analysis (Figure 3) groups the TIR in *Nematostella *NvTLR-1 with its fly and human counterparts, with strong bootstrap support. Surprisingly, three more of the predicted *Nematostella *TIR proteins also contain multiple immunoglobulin (Ig) domains (Figure 2), and thus reflect the domain structure of mammalian interleukin 1 receptors (IL-1Rs). NvIL-1R1 and NvIL-1R2 each contain three Ig domains, and NvIL-1R3 contains two predicted Ig domains (Figure 2) but may be incomplete. In the phylogenetic analysis based on TIR domains the *Nematostella *IL-1R-like proteins form a clade distinct from both the MyD88 and Toll/TLR types (Figure 3), although these cnidarian TIRs appear to be distinct from those in the vertebrate IL-1Rs (data not shown). Several other TIR proteins were detected amongst the sequences of *Nematostella *(Additional data file 1), but were not subjected to further analysis as the TIR domains were incomplete or the sequences were judged likely to be artifactual. Two complete TIRs were identified by searching the available coral datasets. The trace archive yielded one TIR from *Acropora palmata *(ApGenomic) and a second was encoded by an *A. millepora *EST (AmTIR-1). These two coral TIRs are most similar to those in the *Nematostella *IL-1R-like proteins (Figure 3), but no linked domains have yet been identified in these cases.

The Müller group recently reported the identification of MyD88 in a demosponge, *Suberites domuncula *[34]. However, whilst phylogenetic analyses clearly grouped the TIR in this sponge sequence with those present in unambiguous MyD88 orthologs (Figure 3), domain searching indicates that the predicted sponge protein may not have a functional DEATH domain.

### The Toll/TLR pathway is ancestral but some components are missing or highly divergent in *Hydra*

**Figure 4:**
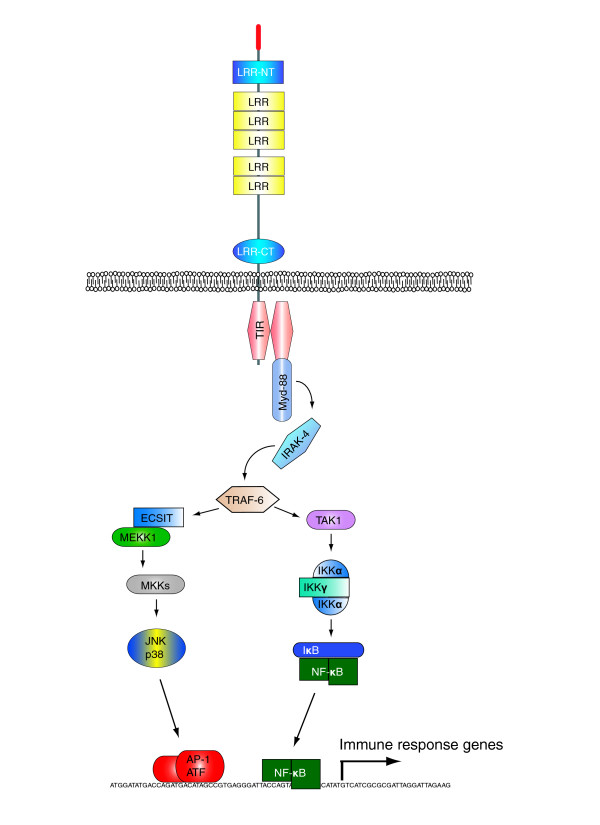
Signaling pathways downstream of the Toll/TLRs. Pattern recognition, either indirectly or directly, by Toll/TLRs results in activation of NF-κB (vertebrates) or the Dif/Rel heterodimer (*Drosophila*) and thus transcription of appropriate immune response genes. At TRAF6, the classic Toll/TIR pathway (shown in the right branch) is linked to the JNK/p38 pathway (shown in the left branch) by the ECSIT protein, which acts as a regulator of MEKK-1 processing [35]. Components of both pathways downstream of Toll/TLRs are represented in the cnidarian datasets (Table 1). ECSIT may also act as a link between these and the TGF-b signaling pathway, since it forms complexes with BMP-pathway restricted Smads and is essential for regulation of the BMP-target gene *Tlx2 *[36]. All of the components of the TGF-b signaling pathway are also known from anthozoan cnidarians [13].

Most of the intracellular mediators of Toll/TLR signaling could be identified in *Nematostella *and *Acropora*, but some key components appear to have either been lost or diverged beyond recognition in *Hydra *(Table 1). The absence of a Toll/TLR protein *sensu stricto *from *Hydra *is discussed above, but in addition only a single highly derived Rel domain could be found in *Hydra *whereas unambiguous NF-κB homologs are present in both *Nematostella *and *Acropora *(Table 1). In addition to the pathway leading to nuclear localization of NF-κB, Toll/TLR signaling can activate the Jun N-terminal kinase (JNK) and p38 mitogen-activated protein kinase (MAPK) pathways, leading to transcription of a range of target genes via the AP1 (activating protein 1)/ATF (activating transcriptionfactor 1) factors. Toll/TLR signaling via JNK/MAPK requires the participation of the ECSIT (evolutionarily conserved signaling intermediate in Toll pathways) adaptor protein [35], which also provides a link between the Toll/TLR and TGF-b (transforming growth factor-beta)/BMP (bone morphogenic protein) pathways [36]. The presence of ECSIT as well as the key components of the JNK/MAPK pathway in the cnidarian datasets (Table 1, Figure 4) indicates an early origin for this variant of Toll/TLR signaling. The discovery of a conserved predicted ECSIT coding sequence in the fresh water sponge *Ephydatia fluviatilis *(N Funayama, personal observation) additionally supports this view.

Wiens *et al*. [34] have suggested that a sponge-specific cell surface protein known as SLIP (sponge LPS-interacting protein) functions as a pattern recognition receptor and effectively substitutes for Toll/TLR in antimicrobial defence. The sponge MyD88-related protein nominally functions downstream of SLIP in the proposed pathway [34]. In support of the idea that sponges lack Toll/TLRs, the authors cite a lack of TLRs in a screen of 15,000 ESTs. As sponge 'MyD88' and SLIP are co-immunoprecipitated by the reciprocal antibodies [34], they clearly can interact *in vitro*. Some TLR pathway components could also be identified in the available sponge data, but the equivocal status of the sponge MyD88 related protein means that it is unclear at this time whether sponges have a canonical Toll/TLR pathway.

### Cnidarian complement C3 and related proteins

**Figure 5:**
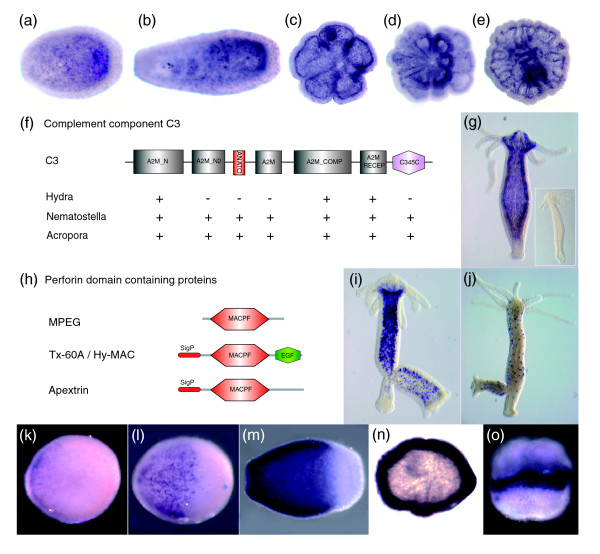
Complement component C3 and MAC/PF domain-containing proteins in Cnidaria. **(a-e) ***In situ *hybridization of C3-Am in *Acropora*. Expression first becomes apparent in scattered endodermal cells concentrated at the aboral end as the planula elongates from sphere to pear (a) and eventually to spindle (b). Endodermal expression continues post-settlement (c-e), becoming especially strong in the upper part of the polyp as it rises from the calcifying base (d). Post-settlement, the polyp consists of a series of hollow chambers interconnected beneath the mouth. The line of strong staining peripherally is the result of viewing the endoderm vertically, while elsewhere one is looking through the staining layer. **(f) **Domain map and presence (+)/absence (-) data for the various protein domains characteristic of complement C3 components in the *Hydra*, *Nematostella *and *Acropora *datasets. **(g) ***In situ *hybridization of the *H. magnipapillata *A2M-related gene. *Hydra *A2M-related transcripts are present in the endoderm along the whole body axis. Note that this *Hydra *gene lacks several of the C3-diagnostic domains that are present in the anthozoan C3s (see text). **(h) **Domain maps of major cnidarian MAC/PF proteins types. **(i) ***Hydra *Tx-60a *in situ*. The insert shows the sense control. **(j) ***Hydra *apextrin *in situ*. **(k-o) ***Acropora *apextrin *in situ*. Expression is first apparent in scattered ectodermal cells orally as the planula begins to elongate (k). At slightly later stages expression has spread toward the aboral end of the planula, still in scattered cells (l). As the elongation process continues, uniform strong expression is localized in all ectodermal cells in the oral two-thirds of the planula (m). The strong ectodermal expression is clearly apparent in this transilluminated transverse section cut from the central region of the planula (n). Following settlement, expression continues at the oral end of the planula, before frequently becoming limited to a narrow ring separating oral and aboral tissue (o).

The complement component C3 has recently been reported in another anthozoan cnidarian, the octocoral *Swiftia *[37], and the corresponding gene has recently been cloned from *Acropora *(Hayward, unpublished data). The *Acropora *C3 (C3-Am) gene is first expressed strongly in the endoderm of the planula as it elongates following gastrulation (Figure 5a). The endodermal expression is not uniform, being most intense in a subset of dark staining cells that have not yet been characterized. As the planula elongates expression becomes somewhat weaker, with the strongest expression localized to the aboral endoderm (Figure 5b). Post-settlement (Figure 5c-e) expression is limited to the endoderm and is particularly strong in the endoderm of the polyp as it rises from the calcifying platform at its base (for example, Figure 5d).

C3 has a complex domain structure. Whilst anthozoan C3s resemble their deuterostome counterparts both in domain structure (Figure 5f) and sequence, not only could no corresponding gene be identified in *Hydra*, but also some of the domains characteristic of C3 (ANATO, C345C; Figure 5f) could not be detected in any Hydra protein. Although lacking a canonical C3, *Hydra *contains a gene encoding A2M related domains. Interestingly, *in situ *hybridization in *Hydra *using a probe covering these typical A2M-related domains (Figure 5f; A2M-comp/A2M-recep) showed expression restricted to the endodermal epithelium (Figure 5g), as was the case with *Acropora *C3.

### MAC/PF domain containing proteins in Cnidaria

Searching for other components of the complement cascade, we identified proteins containing a membrane attack complex/perforin domain (MAC/PF) similar to that present in complement component C6 and related proteins. HMM searching identified just two MAC/PF domain-containing proteins in *Hydra *(Table 1), whereas four proteins were identified in *Nematostella*. Two MAC/PF proteins were also identified amongst the *Acropora *ESTs. Database searches and analyses of predicted domain structures revealed that most of the cnidarian MAC/PF sequences are likely to fall into three groups corresponding to the known proteins types MPEG, TX-60A and apextrin (Table 1, Figure 5h).

TBlastN-based searches of the *Nematostella *genome identified a gene matching strongly to the human macrophage expressed protein 1 (MPEG1; GenBank:XP_166227) and its abalone homolog abMPEG1 (GenBank:AAR82936) [38]. A clearly related gene in *S. domuncula *has recently been implicated as an effector in a hypothetical sponge innate immune defence pathway [34]. Recombinant *Suberites *MPEG has anti-bacterial activity against Gram-negative bacteria, and is up-regulated after lipopolysaccharide (LPS) treatment [34]. The MPEG1 family clearly has an ancient evolutionary history (the sponge and human sequences have 28% identity and 46% similarity) [34] but only in *Suberites *has any functional characterization been done. Despite the presence of MPEG1 in the sponge and an anthozoan, no corresponding gene could be identified in *Hydra*.

The nematocyst venom of at least some anthozoans contains the protein TX-60A [39], and two of the *Nematostella *MAC/PF proteins and one of the *Acropora *ESTs clearly correspond to this protein type (Table 1). TX-60A has an epidermal growth factor (EGF) domain immediately carboxy-terminal of the MAC/PF domain. In *Hydra*, this domain structure can be found in Hy-MAC, one of the two Hydra MAC/PF proteins (Figure 5h, Table 1). However, it is unclear whether the *Hydra *and anthozoan sequences are orthologous, as overall sequence identity is low. *In situ *hybridization analysis shows that expression of Hy-MAC is restricted to gland cells that are interspersed throughout the endoderm of *Hydra *(Figure 5i). Since endodermal gland cells and nematocysts are terminally differentiated [40], this pattern of expression is not easy to reconcile with a common function for the venom TX-60A and Hy-MAC.

### Apextrin, a gene lost from *Nematostella*

The third class of cnidarian MAC/PF proteins represented in the *Hydra *and *Acropora *ESTs (Figure 5h) contains no identifiable domains other than MAC/PF. These proteins have moderate overall similarity to the echinoderm apextrins [41,42] and to the apicomplexan protein family to which the *Plasmodium *membrane attack ookinete protein (MAOP) [43] belongs. MAOP is responsible for rupture of epithelial cells in the insect host by the ookinete stage of the parasite. Surprisingly, apextrin seems to be a case of gene loss from *Nematostella *as, despite clearly related genes being present in *Hydra *and *Acropora*, extensive searching of both the predicted protein collection and the anemone genome using a variety of tools failed to identify an apextrin-related gene (Table 1).

To explore the significance of this case of apparent gene loss, the expression pattern of an apextrin gene was examined by *in situ *hybridization in *Acropora *(Figure 5k-o). *Apextrin-Am *expression first appears in scattered ectodermal cells at the oral (blastopore) end of the embryo as it begins to elongate following blastopore closure (Figure 5k). As the embryo continues to elongate expression increases in intensity and the zone of expression spreads toward the aboral end, initially still in scattered cells (Figure 5l), but as elongation continues apparently in all ectodermal cells (Figure 5m), as is clear in transverse section (Figure 5n). As the planula settles, expression becomes less obvious at the oral end, eventually becoming limited to a belt marking the transition between the tissue that was formerly aboral (bottom), and the end bearing the oral pore, which subsequently forms the mouth of the polyp (Figure 5o). These expression data suggest that the primary function of apextrin-Am is in the ectoderm leading up to metamorphosis; hence, secondary loss of the corresponding gene from *Nematostella *may be explicable in terms of the very different modes of development of these two animals (see Discussion).

In adult *Hydra*, whole mount *in situ *hybridization showed an expression of the apextrin-like gene in groups of ectodermal cells arising from the interstitial cell lineage (Figure 5j), which may reflect a possible functional shift in an organism where metamorphosis is absent.

In addition to the above, the *Nematostella *dataset yielded MAC/PF domain-containing proteins having high similarity to the neural cell adhesion molecule spondin-1 (Additional data file 1).

## Discussion

These preliminary analyses of the newly available genomic and EST datasets indicate that a surprising number of key components of the innate immune system, including the Toll/TLR pathway and some complement cascade components, were in place at the base of the Eumetazoa. Also represented in the datasets are proteins strongly matching both the tumor necrosis factor (TNF) and Nod-like receptors and with the same domain structures (data not shown).

The analyses presented here are consistent with the idea of a genetically complex common metazoan ancestor [12,13]; clearly the Toll/TLR, MyD88 and IL-1R protein families were distinct prior to the divergence of the Cnidaria from the Bilateria, and a Toll/TLR pathway may predate even the Porifera/Eumetazoa split. The discovery of proteins with the same domain structure as the IL-1R in *Nematostella *indicates that this receptor type predates chordate origins and that its original ligands may not have been interleukins. However, as the TIR domains in the cnidarian IL-1R-like and mammalian IL-1R proteins are divergent, separate evolutionary origins cannot yet be ruled out despite the similarities at the level of domain structure.

It is also likely that a prototypic complement effector pathway involving C3 and multiple MAC/PF proteins was in place in Ureumetazoa. Comparisons of the TIR and MAC/PF complements of *Hydra *and *Nematostella *highlight the likely extent of gene loss and sequence divergence in the former - not only does *Hydra *appear to have lost the Toll-receptor, but NF-κB has also either been lost or has diverged beyond recognition. Moreover, only a few TIR proteins could be identified in *Hydra *compared to the rich representation of these in the anthozoans. In addition, Hydra appears to have lost a number of MAC/PF proteins and lacks an equivalent of the ancestral complement C3 protein, implying that although anthozoans most likely have a prototype complement effector pathway, this has undergone degeneration in *Hydra*. *Hydra *is an efficient producer of a number of potent antimicrobial peptides (Bosch, unpublished data), indicating that at least some of the functions of the complement effector system may have been subsumed by pathways involved in synthesis of these peptides.

The observation that genes of two of the three classes of cnidarian immune-repertoire genes examined here are expressed in the endoderm (Figure 2) was unexpected, but is consistent with the primary sites of expression of such genes in many animals. In *Drosophila*, the fat body (an endodermal derivative, and the functional homolog of the mammalian liver) is the predominant source of the antimicrobial peptides whose synthesis is under the control of the Toll and Imd pathways [44] and in *Caenorhabditis *the gut is the primary source of antimicrobials [45,46]. Although mammalian skin contains immune sensory cells (Langerhans cells, dendritic cells and so on), these are also endodermal derivatives, hence there is a deep evolutionary link between the immune system and the endoderm.

Other specific cases of gene loss from *Hydra *have been documented [47] (Seneca, unpublished data), hence there are precedents for the apparent loss of immune repertoire components reported here. More surprising is the implication that gene losses may also have occurred in *Nematostella*, as evidenced by the apparent absence of the apextrin gene that is present in both *Hydra *and *Acropora*. This loss may be associated with differences between *Nematostella *and *Acropora *during the planula to polyp transition. Metamorphosis from the motile planula to the sessile polyp involves dramatic tissue remodeling [48] and gene expression changes in *Acropora *(Grasso, unpublished data), and the massive up-regulation of apextrin leading up to and during metamorphosis (Figure 5k-o) is consistent with a role in this process. It is worthy of note that apextrin is expressed in the tissue that is least remodeled at the time of metamorphosis. By contrast, it appears that there are no dramatic events comparable to *Acropora *metamorphosis occurring during this same period of *Nematostella *development; rather, *Nematostella *undergoes a simple and continuous transition from planula to polyp and appears to show some neotenous features, continuing to glide over the bottom aboral end first for some time after metamorphosis [49], and never forming a pedal disc [50]. Thus, loss of this gene may be related to the different modes of development of these two animals. This comparison between two anthozoans with different modes of development is clearly reminiscent of the original identification of apextrin as a gene specifically expressed in the 'direct' developing sea-urchin *Heliocidaris erythrogramma *during metamorphosis, but which is not expressed during the development of *H. tuberculata*, a congeneric 'indirect' developer [41,42]. Because indirect development is considered to be ancestral in sea urchins, these authors hypothesized that apextrin had been coopted to function in metamorphosis in *H. erythrogramma*. The specific ectodermal expression of homologous proteins during metamorphosis in a cnidarian and an echinoderm is either a remarkable example of convergence or reflects conservation of function at some level. This latter possibility carries with it the implication that the 'simple' continuous planula to polyp transition seen in *Nematostella *might represent a derived state, and that the more dramatic metamorphosis seen in *Acropora *might more closely reflect the ancestral condition.

## Conclusion

An important general implication that flows from these data is that gene loss may occur stochastically. If the genes in a pathway function only in that pathway, then one might expect that entire pathways would disappear following loss of one key component. However, the *Hydra *data appear to contradict this; most of the intracellular intermediates of Toll/TLR signaling are present despite loss of all of the major receptor types (both Toll/TLR and the IL-1R types known from more basal cnidarians are apparently absent from *Hydra*). Moreover, simple comparisons between related animals (for example, *Nematostella *versus *Acropora*) are unlikely to be informative in terms of understanding the origins of genes [50] - loss of a gene from *Nematostella *and loss of a specific function in the indirect developing urchin seems more likely than the independent co-option of the same protein to related roles in *Acropora *and a direct developing urchin. Stochastic loss underlying the distribution patterns of genes across the Metazoa may account for some cases of assumed lateral gene transfer - chance retention of an ancestral gene in one or a few animal lineages might easily be confused with lateral transfer. Reconstructing the genome of the common animal ancestor will not, therefore, be a simple undertaking; it will require whole genome data for a wide range of 'lower' as well as 'higher' animals.

## Materials and methods

### Datasets

Genomic and EST sequence data were downloaded from public databases at NCBI (dbEST, Trace archive) or originate from private investigators (D Miller, K Agata) and were stored on a local comparative genomics analysis platform [51]. The raw datasets included 10,171,402 genomic reads and 163,221 ESTs for *H. magnipapillata*, 5,996,730 genomic reads and 146,976 ESTs for *N. vectensis*, 14,625 genomic reads and 10,232 ESTs for *A. millepora*, 11,025 genomic reads for *A. palmata *and 11,450 genomic reads for *Porites lobata*. A set of 36,820 ESTs from the fresh water sponge *E. fluviatilis *is maintained in the lab of K Agata. For the corals *A. millepora*, *A. palmata *and *P. lobata *only pilot genomic sequencing data are available to date. Genomic trace data were maintained in the original raw format, whereas ESTs were clustered and assembled using TGICL tools from TIGR [52]. The ESTscan software [53] was used to infer unigene and predicted peptide sequences from the assembled ESTs.

### Database search, sequence analysis and phylogenetic methods

For database searches a local Blast-platform [51] and the public Blast platform at NCBI [54] were used. Domain searches were performed using SMART [55] and a local install of HMMer [56]. Profile HMMs for the investigated domains were obtained from PFAM [57] and Superfamily [58] databases. Seqtools [59] and BioEdit [60] were used for general sequence analysis. Protein sequence alignments were created using ClustalW [61] and the HMMalign script included in the HMMer package. Maximum likelihood phylogenetic analyses were undertaken using MolPhy version 2.3 [62] using the Dayhoff substitution matrix and local rearrangement search mode.

### *In situ *hybridization

Whole mount *in situ *hybridization on *Hydra *polyps was performed as previously described by Grens *et al*. [63]. *Acropora *embryos were fixed and processed for *in situ *hybridization as described in de Jong *et al*. [64] following the detailed *in situ *protocol given in Hayward *et al*. [65]. For all genes shown, sense controls gave no hybridization signals.

## Additional data files

The following additional data are available with the online version of this paper. Additional data file lists accession numbers for additional sequences identified within the database searches that were not further characterized in the present study.

## Supplementary Material

Additional data file 1 Accession numbers for additional sequences identified within the database searches that were not further characterized in the present study Click here for file

## Figures and Tables

**Table 1:** Overview of innate immunity components present or absent in selected Cnidaria

	**Anthozoa**						**Hydrozoa**		
		
	*Nematostella*			*Acropora*			*Hydra*		
			
		Accession no.	e-value		Accession no.	e-value		Accession no.	e-value
**TLR pathway**									
LBP	+	gnl|ti|1139929806	7e-51	ND			+	gb|DT619160	2e-13
CD14	-			ND			-		
TLR	+	gnl|ti|573160901 gnl|ti|566578628 gnl|ti|558319530 gnl|ti|567085258 gnl|ti|581064934	1e-47	+	gb|EF090256	2e-7	-		
MyD88	+	gnl|ti|1139972660	4e-26	ND			+	gb|CV182656	1e-18
IRAK	+	gnl|ti|1146119691	3e-14	ND			+	gb|DT608600	2e-10
TRAF6	+	gnl|ti|1135509399	2e-51	+	gb|DY583189	1e-38	+	gb|CV985667	3e-41
TAK1	+	gnl|ti|1135635219	1e-51	+	gb|DY583694	8e-119	+	gb|DN812953	1e-45
IκK	+	gnl|ti|1135636054	5e-68	ND			+	gb|CV985420	2e-60
NF-κB	+	gnl|ti|1139960940	1e-74	+	gb|DY582971	3e-36	-		
									
**IFN pathway**									
TRAM	+	gnl|ti|1139940977	9e-66	+	gb|DY579224	5e-72	+	gb|DT615400	1e-58
TRIF	+	gnl|ti|1139933368	4e-07	ND			?		
TBK-1	?			ND			?		
IRF3	+	gnl|ti|1146121907	6e-13	ND			+	gb|DT609518	2e-14
p65	-			ND			-		
IFN-β	-			ND			-		
									
**ECSIT pathway**									
ECSIT	+	gnl|ti|1139978500	4e-35	ND			+	gnl|ti|1223628732	2e-18
MEKK1	+	gnl|ti|1139956887	2e-28	+	gb|DY581138	3e-83	+	gnl|ti|1226566543	3e-25
MKKs	+	gnl|ti|557758729	1e-14	ND			+	gnl|ti|1121918104	1e-18
JNK	+	gnl|ti|1135503269	1e-106	ND			+	gnl|ti|877334588	2e-33
p38	+	gnl|ti|1139959014	1e-114	+	gb|DY579712	5e-111	+	gnl|ti|686048504	7e-39
AP1	+	gnl|ti|1139792930	3e-10	+	gb|DY581320	3e-09	+	gb|CX771032	7e-10
ATF	+	gnl|ti|1139796564	4e-11	ND			+	gb|CN624618	3e-06
									
**Other TLR related proteins**									
HyTRR-1	-			ND			+	gb|DQ449929	0
HyTRR-2	-			ND			+	gb|DQ449930	0
									
**IL1-R related proteins**									
IL1R-1	+	gnl|ti|573182253	0	ND			**-**		
IL1R-2	+	gnl|ti|557993643	0	ND			**-**		
IL1R-3	+	gnl|ti|567060226	0	ND			**-**		
									
**Complement system related proteins**									
C3/A2M related	+	gnl|ti|557724205 gnl|ti|559738307 gnl|ti|558391450 gnl|ti|573218050 gnl|ti|558266068 gnl|ti|573218146 gnl|ti|586367083 gnl|ti|557912603 gnl|ti|573084165	1e-84	+	gb|EF090257	1e-134	+	gb|DT618439 gb|CN554187 gb|CO376061	
C6/C7/C8	-			ND			-		
									
**MAC/PF domain-containing proteins**									
Apextrins	-			+	gb|EF091848	6e-15	+	gb|CV185005 gb|DT613346 gb|CF655657 gb|DT620043	4e-04
Tx60-A	+	gnl|ti|1139936806 gb|DY579588	7e-48 3e-35	+	gb|DY579588	9e-48	+	gb|CV464226 gb|CD680300 gb|BP512716 gb|CV464282 gb|DN246811	1e-07
MPEG	+	gnl|ti|613559286	5e-59	ND			-		

Plus or minus indicate presence or absence of genes, respectively; components marked 'ND' could not be determined within the limited available *Acropora *dataset; question marks indicate not resolvable Blast results, mostly within kinase domain encoding sequences. All accession numbers originated either from GenBank (gb) or from NCBI trace archive (gnl|ti). The given e-values were obtained by BlastX searches against the NCBI nr protein database.
