# Supplementary material for: The innate immune repertoire in Cnidaria - ancestral complexity and stochastic gene loss
Source: Genome Biol. 2007 Apr 16;8(4):R59. doi: 10.1186/gb-2007-8-4-r59 (PMC1896004; doi:10.1186/gb-2007-8-4-r59)
Supplement: Additional data file 1 — Accession numbers for additional sequences identified within the database searches that were not further characterized in the present study [file gb-2007-8-4-r59-S1.doc]

| **Additional sequences identified but not further investigated:** | **Accn.#** |
| --- | --- |
|  |  |
| **Nematostella TIR-domain encoding sequences:** |  |
| predicted protein encoding 2 TIR domains | gnl|ti|613621229 |
| predicted protein encoding 1 TIR domain and 2 ARM domains | gnl|ti|595419898 |
| predicted protein encoding 1 TIR, 2 TM, 1 ANK and 3 Ig domains | gnl|ti|571936680 |
|  |  |
| **Acropora palmata TIR-domain encoding sequences:** |  |
| predicted protein similar to Nematostella IL-1R like protein | gnl|ti|824028928 |
|  | gnl|ti|824031090 |
|  |  |
| **Nematostella MAC/PF domain encoding sequences:** |  |
| predicted protein encoding a putative homolog of the spondin gene (MAC/PF and Spondin domain) | gnl|ti|557738010 |
|  |  |

Supplementary Table 1, Miller et al.
